# The World Health Organization, Corporate Power, and the Prevention and Management of Conflicts of Interest in Nutrition Policy Comment on "Towards Preventing and Managing Conflict of Interest in Nutrition Policy? An Analysis of Submissions to a Consultation on a Draft WHO Tool"

**DOI:** 10.34172/ijhpm.2020.156

**Published:** 2020-08-22

**Authors:** Gary Jonas Fooks, Charlotte Godziewski

**Affiliations:** Department of Sociology and Policy, Aston University, Birmingham, UK.

**Keywords:** Commercial Determinants, Conflicts of Interest, Nutrition Policy, Industry Influence, Corporate Power

## Abstract

The World Health Organization’s (WHO’s) draft Decision-Making Process and Tool to assist governments in preventing and managing conflicts of interest in nutrition policy marks a step-change in WHO thinking on large corporations and nutrition policy. If followed closely it stands to revolutionise business-government relations in nutrition policy. Ralston and colleagues outline how the food and beverage industry have argued against the decision-making tool. This commentary expands on their study by setting industry framing within a broader analysis of corporate power and explores the challenges in managing industry influence in nutrition policy. The commentary examines how the food and beverage industry’s collaboration and partnership agenda seeks to shape how policy problems and solutions are interpreted and acted on and explores how this agenda and their efforts to define conflicts of interest effectively represent non-policy programmes. More generally, we point to the difficulties that member states will face in adopting the tool and highlight the importance of considering the central role of transnational food and beverage companies in contemporary economies to managing their influence in nutrition policy.

The World Health Organization’s (WHO’s) draft *Decision-Making Process and Tool*^1^ to assist governments in preventing and managing conflicts of interest in nutrition policy^1,2^ marks a step-change in WHO thinking on large corporations and nutrition policy as well as an implied acknowledgement that the commodification of food systems by large transnational corporations is driving the global epidemic of diet-related diseases.^3-9^ The decision-making tool encompasses a six-step process of risk assessment, balancing and mitigation.^1,10,11^ It applies generally to non-state actors and is presented as a resource that ‘governments may decide, at their discretion, to follow…completely or partially.’^1^ However, if followed closely it stands to revolutionise business-government relations in nutrition policy in much the same way as Article 5.3 of the WHO Framework Convention on Tobacco Control (FCTC), which aims to protect tobacco policies from tobacco industry political influence.^10,12^

The potential policy implications of the decision-making tool are illustrated by ‘exclusionary criteria’ – outlined in Step Two – to determine whether governments should engage with a non-state actor. These include whether the actor aims to participate in ‘policy development’ – which is broadly defined to encompass agenda setting, policy formulation and decision-making - or ‘contribute (in-kind or financially) to activities related to government normative work or public officials.’^1^ In addition, Step Two provides a ‘risk-based matrix’ which effectively recommends that governments should not engage in ‘high risk’ engagement with corporations that produce just one product for which national dietary guidelines recommend a reduction. Examples of high-risk engagement outlined in an appendix to the tool are extensive and include: those taking place during policy development, monitoring and evaluation; health promotion campaigns; those occurring under conditions of ‘low visibility’; and financial contributions where ‘the external actor advertises its contribution in its promotional material to promote its image.’^1^ The message is clear. Direct and indirect efforts by food and beverage corporations to influence government policy aimed at improving population diets should now be considered major obstacles to reducing diet-related diseases.

## Corporate Social Responsibility and Discursive Power in Nutrition Policy

Ralston and colleagues’ scholarly study of the 2017 WHO consultation on the decision-making tool provides a fascinating account of business thinking on nutrition policy.^10^ The take home message of the paper – that all commercial actors were highly critical of the tool – is instructive, if unsurprising. More importantly, their analysis of the policy frames used by commercial actors provides an important guide to the discursive architecture that underpins contemporary corporate political activity within nutrition policy.

Ralston and colleagues note that commercial actors articulated a ‘collaboration and partnership’ frame which was strongly critical of the tool as exclusionary, viewed its recommendations as inconsistent with principles of good governance, and presented conflicts of interest as compatible with extensive engagement and adequately addressed by existing practices.^10^ The key to understanding this frame and, more generally, commercial actors’ submissions to consultations is that they typically draw on a broader, logically coherent, integrated system of concepts, ideas, and beliefs. Collectively, these constitute a discursive framework advocating a programme and style of governance consistent with strong corporate earnings^13-15^ and represent an important building block of corporations’ ‘discursive power’ in so far as they seek to shape how policy problems and solutions are interpreted and acted on.^16,17^ The framework is carefully developed within corporations’ government and regulatory affairs departments and forms the discursive spine of corporate social responsibility programmes.^14^ In most cases, it represents a heavily socialised rationalisation of corporate interests, which presents a vision of private interests working to produce ‘public goods’ and is normative, ethical and moral in tone and content.

More importantly for understanding the significance of Ralston and colleagues’ work, the ideas and concepts that make up the framework often exhibit one or more of the following characteristics. *Elasticity of Meaning *First, they are often flexible and elastic, having been purposefully developed to be understood differently by actors with different interests – government officials, civil society actors, the general public, shareholders, and corporate partners.^14,18,19^ *The Defensive-Offensive Pirouette *Second, they aim to translate what are essentially self-serving, defensive political positions into constructive, forward-looking proposals, which although resonating with widely accepted social and political values^14^ seek to limit government intervention in markets. *Issue Definition and Appropriation *Third, they seek to appropriate and, ultimately, redefine progressive ideas, concepts, and policy proposals which aim to facilitate ‘public goods’ so that they are more closely aligned with corporate interests.^20,21^ *Non-action through claims of complexity (of the policy problem) and overreach (of the policy solution) *Fourth, they commonly point to the inherent complexity of the policy problem under consideration, which sets up policy proposals as either oversimplified or overreaching and provides a basis for advocating inaction or limited action.^13,22^

## Collaboration, Partnership, and (Non)Decision-Making

In general terms, commercial actors’ strong support for ‘collaboration and partnership’ illustrates the first two of these characteristics. As Hawkes and Buse have noted, partnership, as used in nutrition policy and practice, comprises a ‘mélange of interactions involving a range of different activities, from education campaigns to joint research activities, and a range of processes and structures for interaction.’^23^ This speaks to the elastic quality of the concept, but also its broader political significance in translating what is effectively a defensive position into a constructive proposition. In addition todescribing a diverse set of practices, partnershiprepresents a means of *symbolically* positioning corporate actors with respect to policy-making. In this sense, and without always being explicit, partnership constitutes a rejection of government-led, evidence-based, population level approaches to managing the market environment for food and beverages in favour of non-evidenced based strategies in which government responsibility for population nutrition is displaced onto epidemiologically compromised corporations. Put simply, partnershipultimately implies and constitutes a *non*policy programme, conveyed through the language of constructive engagement.

The *defensive-offensive pirouette* is captured perfectly in Food Industry Asia’s (FIA) submission. As Ralston and colleagues’ note, the FIA called on the WHO to rethink the tool to ensure that the private sector was not ‘shut out…from any meaningful policy discussion,’ reflecting a broader concern that it was ‘formulated in a way that contradicts and would discourage a very wide range of engagement with non-State Actors.’^24^ Their proposed alternative - ‘multi-stakeholder partnerships’ and ‘working collaboratively with government, policy-makers, academics and civil society’ – was presented as a ‘cost-effective mechanism for delivering positive socio-economic outcomes.’^24^ By way of an example, FIA pointed to self-regulatory measures aimed at promoting responsible marketing to children within a ‘framework in which robust *industry-led* standards can be easily incorporated in regional and national regulatory policies.’^24^ In the context of nutrition policy, there is strong evidence to suggest that such measures are severely limited^25-29^ and there is compelling evidence across policy domains, including nutrition, to suggest that they are designed to scotch the introduction of public regulation by effectively filling regulatory space.^30-32^ Despite these well-documented weaknesses, by appealing to the idea that stakeholders with opposing interests can work towards a common goal, multi-stakeholder partnerships are not only presented as efficient, but also above the factious trade-offs of traditional politics. Doing so establishes ‘collaboration and partnership’ as the sensible default position against which dissenting opinions are unfavourably compared and caricatured.

## Conflicts of Interest, Issue Definition, and Non-action Through Claims of Complexity and Overreach

The WHO’s efforts to outline recommendations to manage corporate influence in nutrition policy and practice in the draft tool involve adapting the concept of ‘institutional conflicts of interest,’ which, in effect, covers circumstances where corporate actors’ interests are not aligned with governments’ public health priorities.^2,33^ Ralston and colleagues’ analysis of commercial actors’ response to this innovation illustrate a confused mix of *issue definition *and* claims to complexity and overreach.*

Commercial actors sought to define conflicts of interest in three respects. First, by *narrowing* the concept to include individually-based conflicts of interests only.Second, by *stretching* the concept to include a range of individual ‘sources of bias.’ Described as ‘extensive and complex’ by the International Dairy Federation, these included ‘cognitive,’ ‘publication,’ ‘political,’ ‘ethical,’ ‘statistical,’ ‘ethical,’ ‘philosophical,’ ‘statements in publications,’ a ‘history of unpaid advisory roles,’ and ‘organisational affiliations.’^34-36^ Third, by drawing *equivalence of effect* between these additional sources of bias and conventional financial conflicts of interests.^35^

Conflating the potential effects of interests and potential sources of bias with conflicts of interests makes conflicts of interest appear so pervasive that they cannot be avoided but only disclosed^37^ and is consistent with commercial actors’ preferred solution to focus on ‘transparent disclosure standards.’^24,38^ More to the point, this attempt to redefine the policy problem provided the basis for a contradictory stew of corporate assertions aimed at encouraging the WHO to fundamentally rethink the tool. These included claims that the draft tool represented an ‘inoperable’^39^ case of overreach by virtue of an ‘extremely restrictive and sweeping definition of “non-aligned” actors,’^24,39^ was incomplete ‘in isolating only one kind of non-state actor,’^38^ and was unwieldy in so far as it would require ‘exceptional amounts of time’ to implement.^40^

## Corporate Power and Nutrition Policy

 The evolution of corporate lobbying from an intermittent, reactive enterprise into a systemic, proactive one is, arguably, the most important political transformation of the last 50 years. The WHO’s decision-making tool on conflicts of interest. represents a necessary, but modest response to this transformation and a recognition that political participation plays out on an unequal landscape. But how effective is the draft tool likely to be in reconfiguring the politics of nutrition policy?

The strength of corporate opposition to the decision-making tool provides a clear indication that food and beverage corporations are likely to contest national governments’ efforts to limit their political influence vigorously. The fact that adoption of the tool is discretionary is, therefore, a major weakness. Article 5.3 of the FCTC, for example, is legally binding on signatories to Convention, but still weakly implemented.^12,41^ Further, the fact that the tool notes that governments may consider following the decision-making process ‘partially’ is premised on a fundamental misunderstanding of how companies seek to influence health policy. Corporate political activity is highly varied,^42,43^ plastic and constantly shifting.^12^ This allows corporations to adapt their efforts to influence policy in response to changing institutional norms and public and political sentiment and, effectively, capitalise on all available political opportunities to build consensus within governments, legislatures, and publics against policy change.^12^

Finally, even if the decision-making tool is followed in its entirety, aligning nutrition policy with public rather than corporate interests may continue to be challenging because of governments’ dependence on food and beverage corporations for employment and revenue generation.^44-48^ This not only underpins the effectiveness of corporations’ political activities, but, more importantly, provides the basis of their structural power, which shapes the range of choices open to governments without the need for corporations to pressurise them directly.^45,49^ Equally, corporations’ investment decisions – a major determinant of future employment and revenue generation – are sensitive to political signals concerning taxes and regulation.^50^ There is, of course, a risk of overplaying the significance of either governments’ economic dependence on food and beverage corporations’ investment decisions or the effects of regulation and taxation on these decisions. In practice, both will vary according to a range of factors. Either way, beverage corporations, in particular, have sought to play on government concerns over both by threatening to withdraw investments in response to proposed sugar taxes^51,52^ and producing specious economic impact statements which exaggerate their economic impacts.^13^

In addition, corporations can reinforce their political power by institutionalising their structural advantage in policy-making. Importantly, transformations in institutional arrangements can reorganise political authority within nutrition policy even where they occur in areas not directly related to health.^53,54^ One way in which this reorganisation of political authority occurs is through the transfer of competencies to supranational institutions, such as the World Trade Organization. Thow and colleagues’ work on the potential role of the Codex Alimentarius Commission in imposing restrictions on national governments’ efforts to introduce front-of-pack nutrition labelling illustrates the point powerfully^55^. The Commission plays an important role in setting standards relevant to the interpretation of the World Trade Organization Agreement on Technical Barriers to Trade. Discussions are currently underway under the auspices of the Codex regarding the potential development of guidance on front-of-pack nutrition labelling. Thow and colleagues’ analysis suggests that the governance structure of the Codex reinforces commercial actors’ structural advantages within these discussions and are progressively shaping how the issue is being deliberated.^55^

 In summary, the draft tool is an encouraging development. However, efforts to manage corporate influence in nutrition policy need to be more ambitious if diet-related diseases are to be addressed effectively. A detailed framework, building on the draft tool, but outlining legally enforceable obligations for both national governments and food and beverage corporations is imperative. Wholesale reform of trade and investment agreements and Better Regulation-style policy instruments, such as regulatory impact assessments, is also required. Beyond that, large transnational food and beverage organisations need to be broken up and new modes of ownership for major commercial entities within the food system should be explored.

## Ethical issues

 Not applicable.

## Competing interests

 Authors declare that they have no competing interests.

## Authors’ contributions

 Both authors contributed to the drafting and revision of the manuscript and approved the final version.
